# Learning Care Pathways Framework: A New Method to Implement, Learn, Replicate, and Scale up Care Pathways for and With the Patient

**DOI:** 10.34172/ijhpm.8517

**Published:** 2025-03-11

**Authors:** Jean-Baptiste Gartner, Célia Lemaire, André Côté

**Affiliations:** ^1^Département de management, Faculté des sciences de l’administration, Université Laval, Québec City, QC, Canada.; ^2^Centre de recherche en gestion des services de santé, Université Laval, Québec City, QC, Canada.; ^3^Centre de recherche de l’Institut Universitaire de Cardio-Pneumologie de Québec, Université Laval, Québec City, QC, Canada.; ^4^Centre de recherche du CHU de Québec, Université Laval, Québec City, QC, Canada.; ^5^VITAM, Centre de recherche en santé durable, Université Laval, Québec City, QC, Canada.; ^6^Centre de recherche du CISSS de Chaudière-Appalaches, Université Laval, Québec City, QC, Canada.; ^7^iaelyon School of Management, Université Lyon 3, Lyon, France.; ^8^Institut Universitaire de France, Paris, France.

**Keywords:** Learning Care Pathways, Patient as Partner, Learning Health Systems, Implementation Research, Implementation Science, Pragmatic Sociology

## Abstract

**Background:**

Although care pathways are a response to the calls for a major change in health system redesign initiatives, very few articles have proposed an implementation method. Indeed, no method exists for large-scale projects of care pathways, as sets of interventions within health systems. Drawing on the systems thinking approach and the pragmatic sociology, we describe the implementation methodology of the Learning Care Pathways (LCP) framework, a method to implement, learn, replicate, and scale up care pathways for and with the patient.

**Methods:**

The LCP was conceptually developed through a series of literature reviews on key methodological concepts. As a comprehensive, theory-informed approach, the LCP emerged by linking implementation strategies, research methods, learning mechanisms and outcomes dimensions aimed at optimising care pathways.

**Results:**

Designed around 13 steps grouped into five phases, this framework provides implementation strategies, research methods and learning mechanisms, including levers for patient involvement. The pre-implementation phase enables the selection of the pilot project’s receiving environment and the design of the project. The implementation phase is designed to co-construct and implement an optimised care pathway based on a scientific analysis of the patient journey, the care pathway perceived by professionals, the care pathway from data and integrating knowledge from international clinical practice guidelines. The post implementation phase aims to demonstrate value creation and set up a learning cycle. The replication phase is designed to repeat the method locally to develop horizontal learning and to evaluate scalability. Finally, the scale up phase aims to repeat the method in other territories to accelerate knowledge creation and develop horizontal and vertical learning.

**Conclusion:**

This framework is of particular interest to policy-makers, healthcare managers, and researchers alike, and must be the subject of several experiments to conduct reproducible research that can lead to national Learning Health Systems (LHS).

## Background

**Key Messages**

**Implications for policy makers**

- The Learning Care Pathways (LCP) framework offers a rigorous step-by-step approach to guide policy-makers, managers, and researchers in the structured implementation of care pathways on a national scale for and with the patient.
- Designed around 13 steps grouped into five main phases, the LCP provides implementation strategies, research methods and Learning Health Systems (LHS) mechanisms to co-construct care pathways, demonstrate value creation, develop learning, replicate the method locally, and scale up at national or even international level.
- The LCP offers a useful method to develop learning cycles, horizontal learning between care pathways, and vertical learning to achieve national LHS.
- Because too many projects fail for lack of method, we believe that this framework is of particular interest to policy-makers, healthcare managers, and researchers alike, and that it must now be the subject of several experiments.

**Implications for the public**

The Learning Care Pathways (LCP) provides a new methodology to implement, learn, replicate, and scale up care pathways for and with the patient. The LCP reinforces the role of the patient in the analysis, optimisation and design of care and services, by developing methods that integrate the patient partnership at every stage. In so doing, this method supports the transformation of professional and organisational practices and learning with a view to better meeting patients’ needs and expectations. Involving patient partners in collecting and analysing data and participating in decision-making goes beyond current frameworks and responds to the need for sensemaking and to an urgent call for patient involvement in reviewing and improving the quality of services. The focus on patient needs and preferences and the patient partnership are at the heart of the framework; the only way to transcend and rethink care delivery in terms of their impact on patient experience and outcomes.

In response to several calls for a major change in health system redesign initiatives to better meet patient expectations and deliver greater quality and social value,^1-5^ which represents one of the most pressing public health challenges of our time,^6^ the World Health Organization and national authorities have published guidelines aimed at improving the quality of services and developing learning mechanisms integrated into the delivery of care.^7-9^ In fact, suboptimal performance and inadequate use of resources persist.^2,10,11^ These problems seem to stem largely from persistent organizational, professional and data silos^12^ leading to disruptions in the continuity of health services,^13^ unnecessary waiting times,^14,15^ defects in the flow of information between episodes,^16^ and the performance of examinations that may be unnecessary.^17^ They come also from difficulties in innovating and integrating evidenced-based knowledge into routine clinical practice.^18^ Changing the vision of healthcare delivery by adopting a care pathway approach is a promising way to achieve sustainable improvements in the healthcare system.^19^ However, despite growing evidence on their impact,^14,20-22^ very few articles have proposed a method for implementing care pathways. To the best of our knowledge, only the 7-phase method^23^ exists, but is limited to the implementation of an isolated care pathway and does not incorporate the latest knowledge in the field. Furthermore, the role of patient involvement is unclear. This is why it is necessary to propose a conceptually grounded scientific method to improve rigour, reproducibility, and comparison of care pathway implementation, as a set of interventions within healthcare systems, integrating interfaces between organisations and actors.

###  Challenges for Care Pathway Implementation

 Care pathways are seen as complex interventions in complex systems,^24^ because the organisation of care delivery is made up of a large number of locally and simultaneously interacting entities.^25^ As an intervention aimed at transforming the organisation of care and professional practices based on evidence-based innovation, their successful implementation mobilises strategies from an implementation science approach.^26-28^ Implementation strategies can be defined as “a systematic intervention process to adopt and integrate evidence-based health innovations into usual care”^29^ and improve the sustainability of change.^30,31^ However, the first challenge lies in the fact that the pre-existing method of care pathway implementation does not incorporate the latest implementation strategies, the learning mechanisms that accelerate knowledge creation, and the replication and scaling phases that enable impacts at scale to be achieved. Another challenge is that implementation science uses numerous models and frameworks to describe, organise, and understand the complexity of changing practice patterns,^32,33^ with a lack of connections between methods, concepts, and theory.^34^ Furthermore, we believe it is essential to go beyond the idea of technical standardisation, and identical reproducibility,^35^ to a mainstreaming approach that recognises the need for flexibility and adaptation^36^ and the need to include a social science approach.^19,36^ Finally, it is recognised that there is a lack of conceptual and practical tools for guiding^37,38^ and assessing implementation,^37,39^ undermining the ability to generalise and exploit results across studies and contexts.^40^ These are the challenges we seek to address in this article, describing the development and implementation methodology of our Learning Care Pathways (LCP) framework.

###  Specification of the Theoretical Foundations of the Learning Health Systems 

 To develop our framework, we drew on two highly compatible theoretical frameworks, the systems thinking approach^41-43^ and the new pragmatic sociology.^44-46^ Indeed, the affinity between them has already been recognised.^47,48^ On the one hand, systems thinking allows developing an holistic view of complex adaptative systems,^49-53^ referring to a number of concepts such as feedback, adaptation, and emergence.^54^ Systems thinking offers promising paradigms for research-practice translation,^55^ focusing on sensemaking^56^ and proposing an adaptive approach that recognises the need to think flexibly, to understand and respond to the local context, and to tailor intervention to best suit different contexts.^57^ On the other hand, the pragmatic sociology supports the analysis and understanding of the transformation of professional practices and collective action.^58^ It enables us to take a precise look at the factors involved in change at individual and organisational levels, considering that individual modes of valuation^45,59^ underpin individual and collective action, thus allowing the change at large scale to be understood.^60^

###  Development of the Learning Health Systems for and With the Patient

 The LCP began out of a recognition that care pathway implementation presents some unique challenges due to its complexity^24,61,62^ and a lack of integration of theory, concepts and methods for effective implementation and comparison.^19^ The LCP is designed and developed using a “patient-as-partner” approach.^63-65^ This approach integrates the patient experience to optimise or redesign service delivery,^25,66-68^ in response to an urgent call for patient involvement in reviewing and improving the quality of services.^69-75^ In addition, the LCP incorporates mechanisms from the Learning Health Systems (LHS) approach^7,76-82^ for learning and engagement of stakeholders such as patients and researchers. Viewing care pathways, as a set of interventions within health systems, the LCP integrates the replication and scaling up phases to fully implement the approach at scale. Based on a series of literature reviews, the LCP was tested and developed during a two-year multidisciplinary research project in the province of Quebec, Canada. The “patient-as-partner” approach is the common thread running through our framework to truly implement care pathways for and with patients.

## Methods

**Figure 1:**
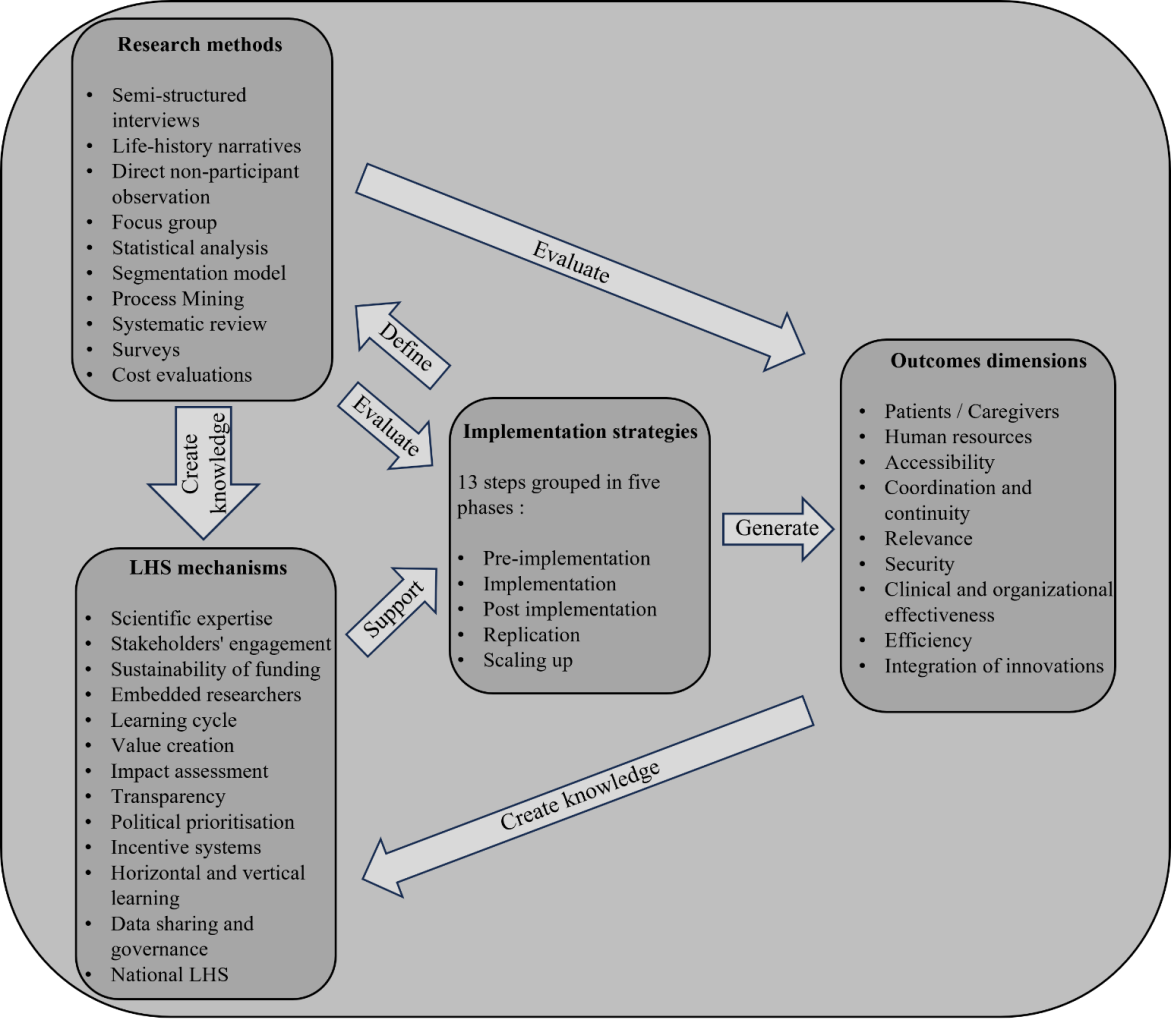
Links Between Implementation Strategies, Research Methods, Learning Health System Mechanisms, and Outcomes Dimensions. Abbreviation: LHS, Learning Health Systems.

 In developing the LCP, we began with a series of literature reviews to cover the field of care pathways. First, we focused on the definition and conceptualisation of care pathways.^19^ Using an innovative hybrid method combining systematic review, concept analysis and bibliometric analysis, we were able to provide a detailed understanding of care pathways and a clear definition for international consensus. In addition, we have formulated attributes, antecedents as success factors and consequences as potential outcomes, linked to their key performance indicators.^19^ We then turned our attention to implementation models for LHS and the learning mechanisms integrated into the models.^83^ Finally, we looked at strategies for implementing innovations and interventions, with an emphasis on replication and scaling up. The developed method has been the subject of several presentations at international congresses^84,85^ and is used in the National Health and Social Services Leadership Development Program of the Ministry of Health and Social Services of Quebec, Canada. Thus, the implementation of the LCP has led to the development of a series of implementation strategies, research methods and learning mechanisms aimed at optimising care pathway outcomes (See Figure 1).

###  Implementation Strategies

 The LCP formulates a set of implementation strategies, based on an in-depth analysis of the literature. These strategies are presented chronologically according to the proposed implementation steps. The LCP mobilises an adaptive approach to the implementation,^50,54^ and a pragmatic sociological approach^44,45,59^ to analyse action and the changes achieved. It involves the constant and ongoing participation of patient partners^63-65^ in project design, strategic governance of implementation, data collection and analysis, development and prioritisation of optimisations, evaluation of results and decision-making. Active participation helps to maintain a common mobilising goal capable of transcending professional and organisational perspectives. But this active participation in all steps of the method is a challenge and needs to be accompanied and supported. To ensure that the vocabulary used is appropriate, we have drawn on the recommendations of the Expert Recommendations for Implementing Change project.^86^

###  Research Methods

 In line with our theoretical positioning combining systems thinking^50,52^ and the new pragmatic sociology,^44,45^ several research methods are mobilised, not only to support implementation strategies through their robustness, but also to develop evidence-based knowledge that enables learning to take place throughout the process. To do so, we suggest mobilising participatory action research methods^87,88^ by involving a group of multidisciplinary embedded researchers throughout the project. Indeed, participatory action research methods are increasingly being used in implementation sciences^89-92^ and from the perspective of pragmatic sociology.^93,94^ In fact, the adoption of evidence-based interventions is more effective when the intervention is internally derived^95^ and is based on scientific and rigorous methods. These research methods are mobilised as part of a “co-learning” approach,^75,91^ as stakeholders are more likely to take ownership of new practices if they are involved in analysing, adapting and implementing the changes. These methods include qualitative methods, such as semi-structured interviews, life-history narratives, observations and focus group, as well as quantitative methods in the form of surveys and statistical analyses, and literature reviews.

###  Learning Health System Mechanisms

 Considering knowledge creation as part of the process, learning mechanisms from the LHS approach are integrated. Indeed, LHS recognises that transforming practices must necessarily be based on both data collection and scientific expertise.^96^ Scientific expertise comes from a multidisciplinary approach, integrating embedded researchers capable of collaborating with the healthcare system to produce novel insights and evidence.^97^ In addition, we mobilise the LHS approach through the implementation of continuous learning through learning cycles.^18,78,98,99^ To reach its full potential, the method must support the development of a strong leadership to scale the approach^100^ and develop a structure capable of supporting horizontal and vertical learning.^7^

###  Outcomes Dimensions

 To assess the success of the implementation and the impact of the optimisations and innovations made, we draw on several methods and frameworks covering a wide range of dimensions integrating both clinical, operational, and organisational indicators. These outcomes dimensions and examples of indicators have emerged both from our systematic review on care pathways^19^ and from evaluation frameworks derived from implementation science and specialised in translating research into practice, such as the Reach, Effectiveness, Adoption, Implementation, and Maintenance (RE-AIM).^101-103^ In fact, the RE-AIM is usually used to systematically assess the robustness of interventions across settings and the potential for scaling up and spreading to additional settings.^103^

 To ensure the completeness of the recommendations, we have included links to existing implementation research frameworks. The Consolidated Framework for Implementation Research (CFIR) supports systems thinking in a multilevel context.^95,104-106^ The CFIR is one of the most cited determinant frameworks that aim to predict or explain barriers and facilitators (determinants) to implementation effectiveness (the outcome).^33,105^ The RE-AIM structures the proposal of the method.^101-103^ The Intervention Scalability Assessment Tool (ISAT)^107^ is used to structure and assess the scalability of the intervention.

 In addition, to ensure the quality and exhaustiveness of the description, we followed the Standards for Reporting Implementation Studies^108,109^ (See Supplementary file 1 for the completed checklists).

## Results

 We have developed the LCP around 13 steps grouped into five main phases. This framework represents an operational methodology that provides information on the targeted activities that will collectively lead to an implementation plan tailored to the local and national, or even international, context. By grouping together implementation strategies, research methods and LHS mechanisms for each step (See Table 1), the LCP offers a roadmap, but is not a one-way process, as the results obtained at one step may influence the other steps, thus proposing an adaptive approach.

**Table 1:** Integration of Implementation Strategies, Research Methods, LHS Mechanisms and Links With Implementation Research Frameworks by Phase of LCP Framework

**Phase**	**Step**	**Implementation Strategies**	**Research Methods**	**LHS Mechanisms**	**Links With Implementation Research Frameworks**
Pre-implementation	1	- Assess tensions conducive to change - Ensure aligned leadership - Assess organisational readiness		- Ensure scientific expertise	CFIR-3 D. Culture CFIR-3 E. Tension for Change CFIR-3 G. Relative Priority CFIR-3 I. Mission Alignment
	2	- Build buy-in (involve governance structures, local champions) - Involve patient partner at strategic level - Build commitment of all stakeholders - Identify barriers and conflicting values - Ensure multidisciplinarity - Develop an adapted communication strategy - Ensure the sustainability of key skills - Anticipate replication and scaling up	- Semi-structured interviews	- Stakeholder’ engagement mechanisms - Anticipate funding mechanism for sustainability	CFIR-1 F. Innovation Complexity CFIR-2 B. Local Attitudes CFIR-2 C. Local Conditions CFIR-2 D. Partnerships & Connections CFIR-2 F. Financing CFIR-3 J. Available Resources CFIR-4 A. High-level Leaders CFIR-4 D. Implementation Facilitators CFIR-4 E. Implementation Leads CFIR-4 F. Implementation Team Members CFIR-4 G. Other Implementation Support CFIR-5 E. Tailoring Strategies
Implementation	3	- Characterise population - Capture patient referral criteria - Identify clusters	- Statistical analysis - Segmentation model	- Patients' medical and socio-demographic data collection	RE-AIM. Reach
	4	- Capture patients' experiential knowledge - Understand structural, organisational, and operational facilitators and barriers - Capture and share local knowledge	- Life-history narratives - Direct non-participant observation - Semi-structured interviews - Process mining - Systematic review	- Qualitative data collection - Patients’ trajectories data collection - Embedded researchers	CFIR-2 E. Policies & Laws CFIR-3 A. Structural Characteristics CFIR-3 B. Relational Connections CFIR-3 C. Communications CFIR-5 F. Engaging
	5	- Establish a shared vision of the need for change - Innovations supported by clinical evidence - Demonstrate potential value creation through simulation - Understand interdependencies	- Triangulation - Gap analysis - Business process modelling - Optimisations simulation	- Knowledge and evidence synthesis - Demonstrate the relevance of changing practices	CFIR-1 B. Innovation Evidence Base CFIR-5 B. Assessing Needs CFIR-5 C. Assessing Context
	6	- Facilitating change at individual and organisational level - Rely on local champions and leaders - Rely on external change agents - Co-design solutions - Maintain the sense of change - Implement changes	- Focus group	- Knowledge translation - The meaning of value creation for the patient	CFIR-1 A. Innovation Source CFIR-1 C. Innovation Relative Advantage CFIR-1 D. Innovation Adaptability CFIR-1 E. Innovation Trialability CFIR-1 G. Innovation Design CFIR-2 G. External Pressure CFIR-3 F. Compatibility CFIR-3 H. Incentive Systems CFIR-3 K. Access to Knowledge & Information CFIR-4 B. Mid-level Leaders CFIR-4 C. Opinion Leaders CFIR-4 H. Innovation Deliverers CFIR-4 I. Innovation Recipients CFIR-5 A. Teaming CFIR-5 D. Planning CFIR-5 G. Doing
Post implementation	7	- Assess impact of care pathway implementation - Demonstrate value creation	- Semi-structured interviews - Pre-post surveys - Cost evaluations	- Impact assessment	CFIR-1 H. Innovation Cost CFIR-5 H. Reflecting & Evaluating RE-AIM. Effectiveness RE-AIM. Adoption – Setting Level RE-AIM. Adoption – Staff Level RE-AIM. Implementation
	8	- Support the monitoring and use of methods - Analyse and document adoption and self-organisation	- Surveys - Indicators monitoring - Cost evaluations - Semi-structured interviews	- Learning cycle initiation	CFIR-5 I. Adapting RE-AIM. Maintenance – Individual Level RE-AIM. Maintenance – Setting Level
Replication	9	- Promote an additive strategy while preparing the multiplicative strategy - Develop a glossary of implementation - Ensure the comparability of assessment dimensions for horizontal learning	- The same as steps 3 to 8	- The same as steps 3 to 8	CFIR-1 D. Innovation Adaptability CFIR-2 A. Critical Incidents CFIR-2 F. Financing CFIR-2 G. External Pressure RE-AIM. Reach RE-AIM. Effectiveness RE-AIM. Adoption – Setting Level RE-AIM. Adoption – Staff Level RE-AIM. Implementation
	10	- Analyse and document self-organisation - Sustain collaborative learning	- Surveys - Indicators monitoring - Cost evaluations	- Horizontal learning initiation - Transparency of data - Knowledge sharing activities	CFIR-5 I. Adapting RE-AIM. Maintenance – Individual Level RE-AIM. Maintenance – Setting Level ISAT A4. Evidence of Effectiveness ISAT A5. Intervention Costs and Benefits
	11	- Assess scalability - Adapt scaling-up strategy to political and environmental context		- Political prioritisation of a learning healthcare system	CFIR-2 F. Financing ISAT A3. Strategic/Political Context
Scaling up	12	- Implement the multiplicative strategy - Develop and organise monitoring systems - Develop control and incentive systems	- The same as steps 3 to 8	- Quality and performance transparency - Incentive systems	CFIR-1 D. Innovation Adaptability CFIR-2 A. Critical Incidents CFIR-2 G. External Pressure RE-AIM. Reach RE-AIM. Effectiveness RE-AIM. Adoption – Setting Level RE-AIM. Adoption – Staff Level RE-AIM. Implementation ISAT B1. Fidelity and Adaptation ISAT B3. Delivery Setting and Workforce
	13	- Create an infrastructure responsible for monitoring and steering care pathways integrating patient partners - Establish a culture of sustainable continuous learning - Ensure the independence and the research base of the infrastructure		- Vertical learning initiation - Data sharing and governance infrastructures - National LHS	CFIR-5 I. Adapting RE-AIM. Maintenance – Individual Level RE-AIM. Maintenance – Setting Level ISAT B2. Reach and Acceptability ISAT B4. Implementation Infrastructure ISAT B5. Sustainability

Abbreviations: CFIR, Consolidated Framework for Implementation Research; ISAT, Intervention Scalability Assessment Tool; LCP, Learning Care Pathways; LHS, Learning Health Systems; RE-AIM, Reach, Effectiveness, Adoption, Implementation, and Maintenance.

###  Phase 1: Pre-implementation

 The pre-implementation phase begins when the opportunity or desire to implement an LCP project is clearly defined and supported by key players of a healthcare system.

####  Step 1: Identify the Receiving Environment of the Pilot Care Pathway

 In this first step, a formal or informal committee of key players from the research community with knowledge and skills in these methods, supported by decision-makers, must analyse all possibilities of the receiving environment. Firstly, it is necessary to assess whether there is a tension conducive to change.^110^ This tension can arise from an awareness of suboptimal practices,^105,111^ coordination problems within networks or between facilities,^111^ or perceptions of inadequacy of care organisation in relation to patient expectations. However, it is necessary to perceive a minimum need to change,^112^ facilitating the opportunity for sensemaking ^56,113^ and paving the way for the potential value creation.^114^ Secondly, it is necessary to ensure that there is an effective, aligned and strong leadership,^110,112,113^ with an ability to target champions and opinion leaders.^115,116^ Finally, it is important to ensure that there is an absorptive capacity^112,113,117^ through organisational slacks, no apparent strong tension, and a culture that encourages integration of new knowledge,^113^ to ensure organisational readiness for implementation.^112,115^

####  Step 2: Design of the Pilot Project

 Once the receiving environment is known, it becomes necessary to create a multidisciplinary group responsible for designing the pilot project, which will enable the necessary resources to be specified. We recommend ensuring multidisciplinary leadership and participation by including academic and clinical researchers, leaders of the receiving environment, two experienced patient partners, a representative of decision-makers and potentially a representative of a technological partner that can support technological innovations.^114^

 The project is designed as a change management project, setting up a steering committee, responsible for the strategic direction and for monitoring, made up essentially of the members of the pilot project’s design group. We recommend assessing expectations and objectives of all team members to identify potential barriers or conflicting values, using semi-structured interviews. This committee is responsible for choosing the main diagnoses to be covered, based on the perceived potential value creation. The diagnoses chosen must be statistically significant to have the most significant impact ie, the percentage of diagnoses per year must be significant compared with the total number of diagnoses in a speciality. We therefore recommend targeting the three to four most frequent diagnoses representing more than 60% of a speciality’s total diagnoses per year. An operational committee is created, bringing together a wide range of skills and capable of mobilising all the necessary methods. The multidisciplinary nature is highly recommended as an over-representation of clinicians exposes to the risk of being too deeply rooted in the clinical organisational culture. The ability to target local clinical champions appears key,^118^ these becoming supporters of the initiative at all levels.^115^ Finally, patient partners are included. In our view, novice patient partners from the receiving environment will be better able to share their experiential knowledge, while gradually increasing their comprehension. Their understanding is crucial for effective participation and the development of confidence, enabling them to intervene in the decision-making process.^119^ One of the keys to success lies in the capacity to develop a team culture that fosters effective communication and to support the relevance of the patient partner’s interventions.

 Finally, the project needs clear support from decision-makers, in terms of policy, time, and resources.^114,115^ Indeed, it is essential to ensure the sustainability of methodological skills and knowledge,^7,120^ and to anticipate the replication and scaling-up phases.^39,121^ Therefore, the funding model should incorporate phased funding from the outset, guaranteeing potential funding for subsequent phases in case of value creation demonstration. At the end of this phase, the decision to launch the pilot project is validated, the target diagnoses defined, the organisation’s readiness assessed, and the necessary resources secured.

###  Phase 2: Implementation

####  Step 3: Define and Characterise the Target Population

 Organising care into pathways requires the ability to assign patients to pathways. It is therefore necessary to characterise the population sufficiently to correctly define the target population for the analysis phase and the selection criteria. Characterisation is based on a statistical analysis of patients’ socio-demographic data and healthcare services data over several years. This analysis should highlight key characteristics as well as associated comorbidities and the existence of subsegments of patients with very different treatment profiles. Using patient segmentation models^122^ by identifying clusters, the aim is to group together patients who share similar clinical needs in order to offer personalised care, based on combinations of interventions or healthcare strategies that best meets their needs^123,124^ and to understand key factors that guide clinical management of patients.

####  Step 4: Analysis of Current Care Pathways and Best Practices

 There is an empirical distinction between three elements of a care pathway, each with its own type of analysis, the care pathway experienced by the patient (ie, the patient journey),^17,125-127^ the one as perceived by professionals and the care pathway resulting from data analysis of patient trajectories.

 Patient journey consists of sequential steps in the clinical process of the patient through their experience, consisting of patients’ interactions with multiple care settings over time.^128^ For the analysis of patient experiences,^17,127,129,130^ interviews with patients must be made using life-history narratives.^131^ This method is crucial for a fuller understanding of phenomena.^132-135^ Then, for portions, direct non-participant observation^136^ are used, where the researcher tries to understand the world, relationships, and interactions in a new way. These qualitative methods allow access to the organisational context and insights into implementation facilitators and barriers.^137^

 The care pathway as perceived by professionals integrates complementary and partial professional perspectives. Its understanding enables a focus on ways of improving the provision of care, the mechanisms of communication and coordination between stakeholders, and the development of interdisciplinarity. Semi-structured interviews should be conducted with all types of professionals involved as well as operational and middle managers, using an interview guide and in the presence of a patient partner who can question the information provided. In addition, direct non-participant observations with a patient partner enable the questioning of professionals in their professional context and during action, highlighting the organisational and structural constraints they face in daily practice.

 Analysis of patients’ trajectory data is a way of highlighting the pre-existing care pathways using process mining.^138-140^ The major difficulty lies in collecting and merging data of varying quality from different databases and organisations. It is essential to carry out data cleansing, removing outliers and managing missing data appropriately. Data mining specialists need to work alongside operations experts and patient partners to ensure consistency between the categories of data available, the results of process mining and operational reality. Combined methods using simulation modelling and machine learning have now proved their worth for designing care pathways.^141,142^

 In addition, unlike most approaches to continuous quality improvement, which remain focused on the current care pathway, it is essential to integrate knowledge from international clinical practice guidelines based on a systematic literature review. Therefore, a fourth dimension is added, the theoretical care pathway, to build on the evidence and accelerate the transformation.

 Finally, it is essential to pay particular attention to power dynamics during data collection and analysis, the kinds of data that are collected, and how and by whom those data are analysed,^36^ and to go back and forth between collecting data, interpreting the research material, and validating the interpretations.

####  Step 5: Synthesis of Operational, Organisational, Social, and International Clinical Knowledge

**Figure 2:**
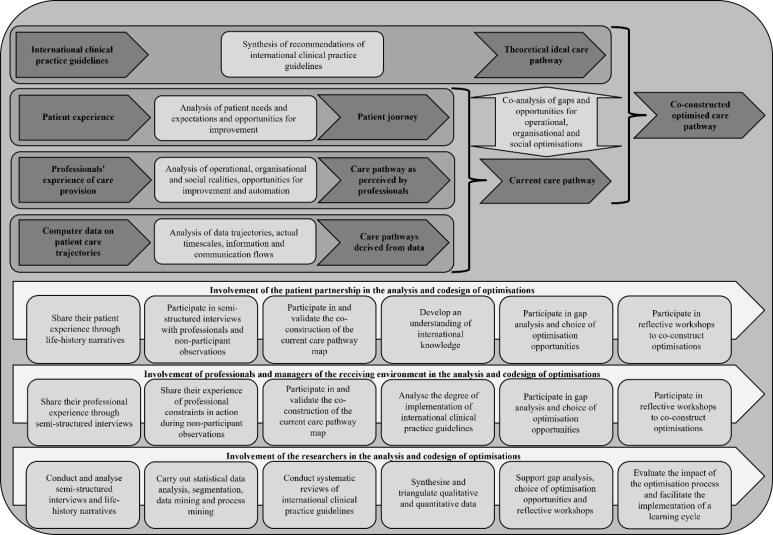
Reference Model for Care Pathway Optimisation for and With the Patient.

 There are two parallel syntheses. On the one hand, synthesis of current care pathway knowledge must be achieved by triangulating data.^143^ Analysis of the patient journey highlights the needs and preferences of patients and focus on specific problems and areas for improvement. The care pathways as perceived by professionals emphasise features of the tasks and activities, organisational and professional practices, but also coordination structures, communication methods, the working climate and information management. Finally, process mining makes it possible to integrate a large part of the knowledge on the preferred pathways, the actual progress of activities including average times, but also efficiency and performance issues. By combining these three components, it is possible to develop a detailed understanding of the patient experience and of operational, organisational, and social realities. On the other hand, the synthesis of clinical practice guidelines provides a list of recommendations drawn up in chronological order of the course of the care pathway. By confronting the theoretical care pathway and current care pathway, a gap analysis is made, formulating a set of clinical recommendations. Similarly, by comparing patients’ needs and preferences with the organisational and social issues, it is possible to formulate operational, organisational, and social recommendations for improvements and innovations. Understanding these four components enables the definition of the optimised care pathway that is desirable (See Figure 2 for integration of the four dimensions), establishing a shared vision of the need for change, which can be accurately modelled. We recommend the use of Business Process Modeling Notation 2.0 and the Decision Modeling Notation,^144-146^ because it provides not only the description of the process and clinical decision support, but also becomes a simulation tool highlighting the nature and strength of certain interdependencies,^56^ as well as supporting automation.

####  Step 6: Implementing and Adapting in the Receiving Environment

 When implementing care pathways, a number of key factors need to be taken into account to avoid non-adoption and abandonment by individuals or organisations.^84,114^

 At individual level, actors have agency and motivation,^96,147^ which is coloured by personal life and values.^44,45,59,147^ It is therefore essential to facilitate change using local champions because they can wield influence on others, but also to use researchers and patient partners as external change agents,^96^ supporting the relevancy of the value creation of the solutions.^147^ Thus, optimisations are co-designed through focus groups with field representatives of all stakeholders and patient partners, recognising the self-organisation capacity^56^ and the wealth of potentially complementary or conflicting perspectives, a major factor in achieving buy-in and context-specific solutions to complex problems. Patient partners help to maintain the creation of sensemaking for all stakeholders,^56^ by the obligation to develop concrete, understandable and explainable solutions that are meaningful for the patient experience of care and outcomes.

 At the organisational and interorganisational levels, particular attention must be paid to the social and organisational dynamics of the entities involved such as the financial and political environment and the broader societal context.^148^ Thus, optimisations need to be appropriate to the context, but also clearly supported by hybrid leaders who bridge the roles of managers and clinicians and the associated rationalities, fostering staff confidence in changing practices.^149^ In fact, they can help to tap into the organisational and societal influences that shape and constrain individuals’ actions.^150^

 At the end of this phase, the co-constructed optimised care pathway (See Figure 2), integrating the involvement of the patient partnership at every step, is known, and contextualised in professional practices.

 The implementation phase concludes with the implementation and adaptation of the clinical, organisational and social optimisations prioritised by the actors involved.

###  Phase 3: Post-implementation

####  Step 7: Impact Assessment/Proof of Value Creation

 To adequately assess the impact of the optimisations made and the success of the implementation, here too we draw on several methods and frameworks.^19,101-103,151,152^ In fact, it is essential to cover a wide range of dimensions integrating both clinical, operational, and organisational indicators.^119^ The direct and indirect benefits relate to nine interrelated areas of care pathways. Indeed, interdependencies must be considered because the creation of value in a dimension could well have a negative impact on others, undermining the proof of value creation (See Table 2). This evaluation uses three methods in parallel: pre-post intervention surveys, semi-structured interviews, and indicators monitoring. Finally, several economic evaluation methods can be combined, such as bottom-up micro-costing of the care pathway^153,154^ or socio-economic analysis of hidden cost.^155-157^ It is necessary to understand how dynamic system changes affect intervention expansion and impact.^158^

**Table 2:** Outcomes Dimensions and Indicators of Direct and Indirect Outcomes

**Outcomes Dimensions**	**Examples of Indicators or Standardised Questionnaires**	**Instrument or Calculation Formula**	**Collection and Follow-up Method**
1. Patients/Caregivers	Patient Reported Experience Measures	PPE-15 survey	Pre-post surveys then annual monitoring
	Patient Reported Outcome Measures	SF36 survey	Pre-post surveys then annual monitoring
	Number of complaints	Total no. per year	Indicator monitoring by department
2. Human resources	Assessments of Psychosocial Job Characteristics	JCQ survey	Pre-post surveys then annual monitoring
	Perceived % time allocated to writing information per day	No. of hours/Total no.	Semi-structured interviews by profession then annual monitoring
	% overtime	No. of overtime hours/Total no.	Indicator monitoring by profession
	Absenteeism rate	No. of days absent/Total no.	Indicator monitoring by profession
	Turnover rate	No. of departures per year/Average no. of employees	Indicator monitoring by department
3. Accessibility	Average time between request and access to a professional or exam	Sum of delays/Total no. of completed requests	Indicator monitoring by profession or exam
	Number of patients on waiting list for hospitalisation, treatment or examen	No. of patients	Indicator monitoring by hospitalisation, treatment or examen
	% inpatients awaiting transfer	No. of awaiting patients/Total no.	Indicator monitoring
	Non-medical cancellation rate for treatment or exam	No. of non-medical cancellation/Total no.	Indicator monitoring per treatment or exam
4. Coordination and continuity	Duplication of clinical information gathered by different professionals	Percentage of identical information captured by two professions without justification	Comparative analysis of completed forms by profession
	On-time processing rate (compliance with standards)	No. of on-time/Total no.	Indicator monitoring
	Proportion of family doctors/rehabilitation services adequately informed	No. of professionals or structure adequately informed/Total no.	Pre-post surveys then annual monitoring
5. Relevance	% patients who receive written information	No. of patients reporting/Total no.	Pre-post surveys then annual monitoring
	Perceived rate of use of decision-support tools (clinical practice guidelines)	No. of professionals reporting use/Total no.	Semi-structured interviews by profession then annual monitoring
6. Security	Number of adverse events	No. of adverse events	Indicator monitoring
	Number of accidents	No. of accidents	Indicator monitoring
	Number of sentinel events	No. of sentinel events	Indicator monitoring
	Prescription compliance rate	No. of prescriptions correctly filled/Total no.	Pre-post audits then annual monitoring
7. Clinical and organisational effectiveness	Average length of stay per diagnostic	Sum of length of stay/Total no. of patients	Indicator monitoring
	Readmission rates at 8 and 30 days	No. of readmission/Total no. of patients	Indicator monitoring
	30-day mortality rate	No. of death within this period/Total no. of patients	Indicator monitoring
	Bed occupancy rate	No. of occupied beds/Total no.	Indicator monitoring
8. Efficiency	Average cost per trajectory for a diagnosis	Total cost/Total no. of patients per diagnosis	
	Hidden costs avoided because of optimisation (waste, loss of time)	Socio-economic analysis	Pre-post analysis
9. Integration of innovations	Trajectory compliance rate regarding care pathway	No. of trajectory following care pathway/Total no.	Pre-post audits then annual monitoring
	Degree of standardisation of information for decision-making purposes	No. of clinical decision points supported by a decision tree/Total no.	Indicator monitoring

####  Step 8: Initiating a Continuous Learning Cycle

**Figure 3:**
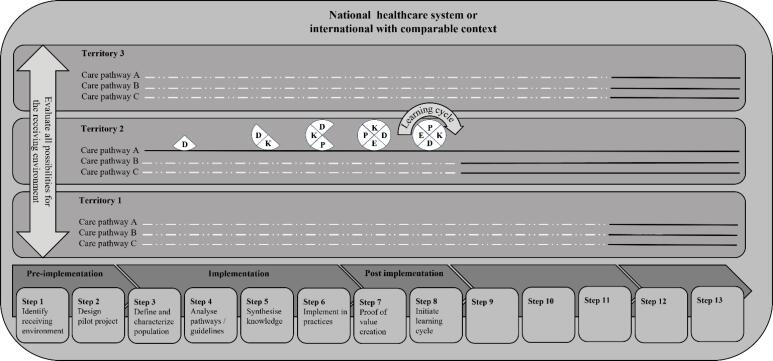
The First Three Phases of the Learning Care Pathways Framework. D = Data: All types of data, qualitative, quantitative, health data, data from computer databases and data from the knowledge synthesis, see Table 2 for all the data involved. K = Knowledge: information or results from data analysis and triangulation. P = Practice: Reflecting the implementation of changes in professional and organisational practices. E = Evaluation: Methods for holistic assessment of the impact of transformation and value creation, enabling a learning cycle to be initiated.

 All too often, care pathway projects stop at the evaluation phase. The LCP sees the holistic evaluation of implementation as an opportunity to initiate and support the receiving environment in implementing a learning cycle required for monitoring and comparison. The implementation of monitoring indicators, surveys, and the training in the use of qualitative methods enable local teams to monitor the evolution of the care pathway. Researchers must support field teams in implementing research methods, reliable and robust monitoring indicators and analysing results. Indeed, the sustainability sought consists of the long-term integration of effective interventions in the receiving environment,^148^ by focusing on acceptability and adoption mechanisms.^159^ In addition, the reach of the intervention within the population cared for by the care pathway must also be monitored.^101-103^ The post implementation phase ends when the receiving environment is able to follow a learning cycle and monitor its results on the outcome dimensions (See Figure 3).

###  Phase 4: Replication

####  Step 9: Replication on Other Care Pathways in the Same Territorial Context

 Once the decision has been made to replicate, based on the proof of value creation and the assurance of support of decision-makers, the methodology can be replicated in the same territorial context. Replication has the advantage of being able to draw on some of the results of the pilot project, but only makes sense if the context is very similar.^160^

 Firstly, replicating the methodology is made easier because not only has the implementation team developed a significant knowledge of the receiving environment, but some of the work will only have to be adapted. Indeed, step 3 is made easier because data collection is already well established. For step 4, the team can capitalise on its understanding of operating modes, action routines and social dynamics, although the understanding of the patient journey and the care pathway emerging from data need to be fully analysed. The systematic review of clinical practice guidelines is also starting from scratch, like the synthesis of knowledge in step 5. The modelling can capitalise greatly on certain modelled subprocesses, which may be common to the care pathways. Finally, step 6 must be carried out in full, adapting optimisations to the context recognising the self-organisation capacity.^56^ The evaluation and implementation of the learning cycle in steps 7 and 8 must draw heavily on previous evaluation methods to enable comparability of results. In addition, although the strategy is rather additive,^112^ ie, the same team of researchers carry out the replication, it makes sense to anticipate the scaling up phase by including stakeholders from other territories to disseminate knowledge in a multiplicative strategy perspective.^112^ Indeed, this phase is an important opportunity to build the belief and will of leaders and frontline staff to support the changes.^149^

####  Step 10: Initiate a Horizontal Learning

 Implementing measures on both qualitative and quantitative data enable contextualised data to be compared and horizontal learning^7^ to be generated, feeding into and enriching the care pathways between them. This can be envisaged if the monitoring retains a high degree of comparability. Standardisation and interoperability of indicators and comparability of themes in qualitative interviews are essential to ensure comparability and reliability. Acceptance of the transparency of data, information, and performance sharing, means that knowledge about the innovations tested can be rapidly created, and solutions can be adapted, appropriated and self-organised, thereby speeding up the process of developing evidence and implementing it in practice.

####  Step 11: Evaluate Scalability

 Scalability assessments^161,162^ must be carried out before the decision to scale up has been made.^163^ Several frameworks for evaluating intervention scalability have been developed,^107,112,113,164^ but the LCP is based on the ISAT.^107^ The experience acquired during the previous steps enables the assessment of the capacity for scale-up and the resources required, taking into account both financial needs and organisational and skill requirements.^112,165^ Scalability assessment must be based on knowledge of the development process of the interventions^107^ and of the strategic, political, or environmental context^107,113,166^ in order to reach a greater proportion of the eligible population.^167^ At this step, the decision is primarily a political one, making it all the more important to take a social approach to change.^113^ This is why it is necessary to rely on available evidence of intervention effectiveness and proof of value creation,^107,112,113^ but also to anticipate known pitfalls.^168^ In fact, the precise knowledge of the costs and skills required are key to the formulation of a scaling-up strategy.^107,112^ The replication phase concludes with the scalability results and a decision on whether to scale up.

###  Phase 5: Scaling up

####  Step 12: Scale the Methodology in Other Territories

 As soon as the decision to scale up has been made and the support of decision-makers assured, the methodology can be scaled up to other territories or countries.^169^ Given the scope and the need to speed up the implementation, it is essential to rely on a multiplicative strategy,^112^ enabling these skills to be disseminated to other territorial teams and making the solution sustainable. Here too, some steps are easier. The summaries of clinical practice guidelines are available and can be simply updated. However, it will always be necessary to give great importance to the operational, organisational, and social particularities of the local context, making it essential to fully implement the LCP and continually adapt the interventions. Thus, adaptation to local context and learning continue to be essential as scaling up proceeds.^112^ However, particular attention must be paid to tackle the infrastructural problems at the time of scaling.^149^ There are several issues that need to be addressed, including political, regulatory, or administrative policies that can either inhibit or accelerate adoption, and the potential use of counteracting strategies such as developing incentives.

####  Step 13: Initiate a Vertical Learning

**Figure 4:**
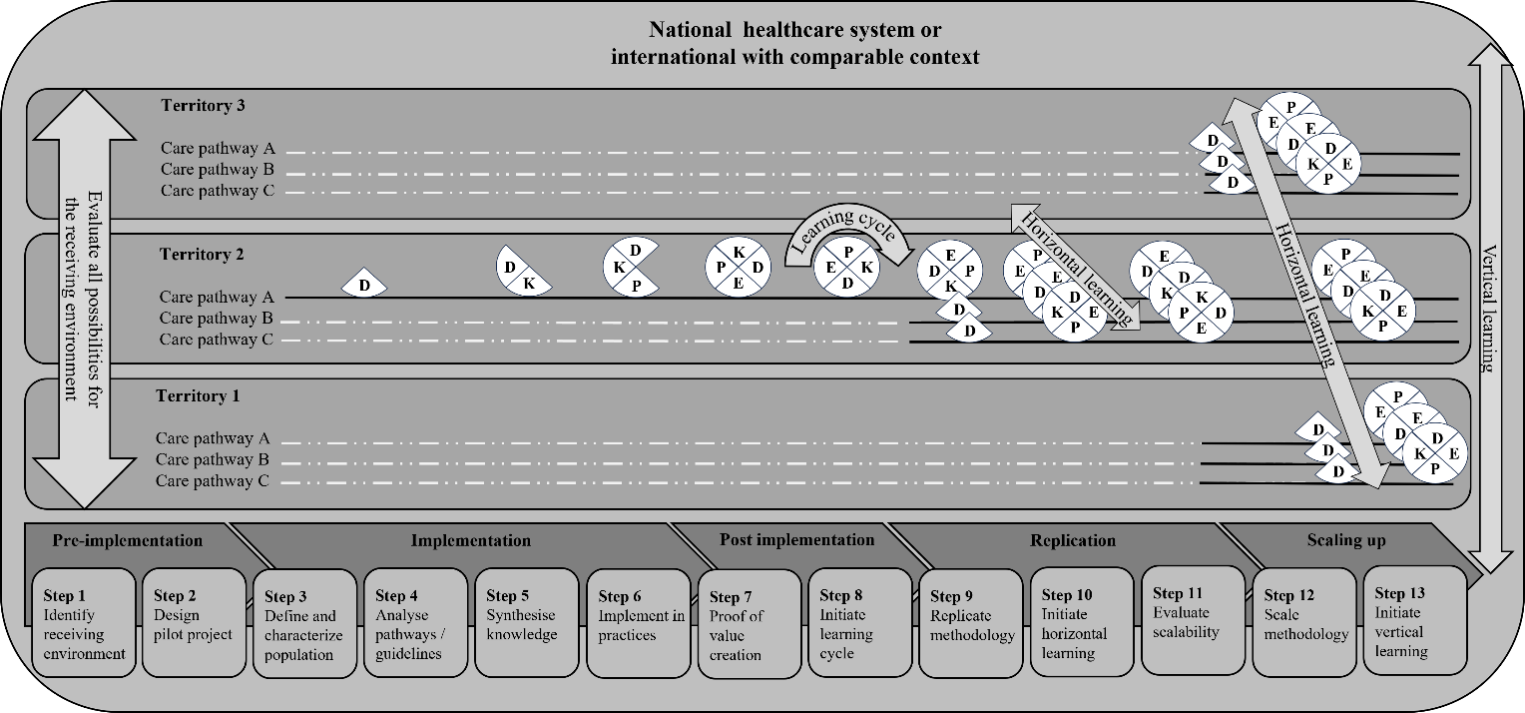
The Learning Care Pathways Framework.

 The thirteenth and final step is designed to set vertical learning^7^ in motion. Indeed, the implementation of learning on care pathways aims to accelerate the creation of knowledge, the demonstration of evidence, the understanding of adaptations to local contexts, and the comparability of care pathways and their results. The implementation of these learning processes must be accompanied by the creation of infrastructure^107^ and resources enabling analysis, understanding, and sharing. Data collection and reporting systems for monitoring can only be implemented if routine data systems are accurate, complete and timely.^149^ This must be facilitated by setting up an entity responsible for monitoring and steering these care pathways at the national level, with the skills and knowledge to support learning and guide decisions. The patient partnership must be active in this infrastructure. However, this entity must be driven by scientific knowledge and have the highest possible level of independence to be sustainable (See Figure 4 for the complete LCP framework).

## Discussion

 In this article, we describe the LCP, a new methodological framework to implement, learn, replicate, and scale up care pathways for and with the patient. This framework responds to the need for guidance on how implementation of care pathways should be managed and sustained to better respond to the patient expectations and to accelerate learning. In fact, for the analysis of existing care pathways, the previous methodological framework^23^ deals only with clinicians’ and managers’ perspectives, data analysis and expert opinions. In addition, it mobilises several analysis methods such as data analysis, interviews and focus groups, based on continuous improvement, without explicitly relying on research methods. Finally, although it includes an evaluation phase, it does not propose a framework for assessing care pathways. In comparison, the LCP clarifies the distinction between three fundamental components of the care pathway analysis: the patient journey, the care pathway as perceived by professionals and the care pathways derived from data. By comparing it with a fourth dimension, the theoretically ideal care pathway derived from clinical practice guidelines, the LCP clarifies the roles of each actor (including patient) involved in the co-construction of the optimised care pathway, as well as the levers which modulate their engagement. Lastly, the LCP offers a framework for the holistic evaluation of care pathways, including outcome dimensions, instruments or calculation formulae and examples of indicators or standardised questionnaires, as well as the collection and follow-up method. The detailed information proposed in the LCP framework brings significant benefits to the implementation of care pathways, as a set of interventions within healthcare systems, integrating interfaces between organisations and actors, an approach for which no method was previously proposed. It also responds to the need for clarity in implementation research^34^ by providing a direct link between implementation strategies, research methods, learning mechanisms and outcomes dimensions. In this way, we believe we are responding to the imperative of relying on a theoretical, conceptual, and practical framework.^164^ Thus, the LCP proposes a practical and actionable roadmap providing recommendations for each step of implementation, replication and scaling up by initiating learning cycles that foster horizontal and vertical learning. In so doing, it goes beyond the technical approach by integrating a social science perspective, which many researchers have called for.^19,36,150,170,171^ In addition, we integrate the perspective of LHS, placing the researcher at the centre of the support for the creation and dissemination of knowledge in practice. The focus on patient needs and preferences and the patient partnership are at the heart of the framework; the only way to transcend and rethink care delivery in terms of their impact on patient experience and outcomes. Involving patient partners in collecting and analysing data and participating in decision-making goes beyond current frameworks and responds to the need for sensemaking,^56,113^ that is necessary for the sustainability of change. The key phase of scaling up is undoubtedly the most difficult to achieve, because political and resourcing factors are often more powerful influences than whether interventions are evidence-based.^166^ Therefore, scale-up remains nonlinear, and is inherently complex and often political.^172^ This is why, the LCP is intended to be highly flexible and adaptable, essentially formulating methods and implementation strategies and must be, to reach its full potential, the subject of clear political prioritisation.^173^ It is important to understand that implementing the LCP should be based on an iterative process including evidence and a continuous self-learning process to achieve the maximum patient-relevant benefits.

 Certainly, the LCP must now be tested and evaluated to establish its value as a generalisable implementation approach. The LCP is currently being deployed as part of a pilot project for three care pathways for chronic obstructive pulmonary disease, pneumonia, and pulmonary fibrosis, in the province of Quebec, Canada. Some of the results of this project on enablers, challenges and barriers to implementing innovations in care pathways have already been published.^174^ It is clear that implementing the model as a whole will require significant investment from healthcare systems over the long term. However, the benefits in terms of expected outcomes and outputs are numerous and the creation of value will need to be demonstrated to justify continued investment. What’s more, the potential for creating value for healthcare systems already exists in the implementation of a single care pathway following only steps 1 to 8.^174^ This is why, the development of pilot projects in several countries would be relevant, allowing us to identify the influence of the social and cultural context on implementation, but also to ensure the reliability and reproducibility of the method. Scaling up would enable participating countries to rapidly develop shared learning and thus further accelerate the creation and sharing of knowledge with a view to better meeting patients’ needs and preferences.

## Conclusion

 The LCP provides a new methodology to implement, learn, replicate, and scale up care pathways for and with the patient. Recognising the complex nature of care pathways and health systems,^19,24,25^ we have attempted to develop a robust methodology that recognises the need to adapt to the local context, the capacity for self-organisation, and exploit the non-linear learning processes inherent in care pathway implementation. Whilst this cannot guarantee the success of any project, application of the LCP’s suite of implementation strategies, scientific methods, and LHS mechanisms, together with the new pragmatic sociology to change,^44,45,59^ at both individual and organisational levels, will support real and lasting transformation in professional and organisational practices, and accelerate learning with the aim of redesigning and optimising the delivery of healthcare services for and with patients. Because too many projects fail for lack of method, we believe that this framework is of particular interest to policy-makers, decision-makers, and researchers alike, and that it must now be the subject of several experiments. In addition, we believe that LCP is one of the possible solutions to implement a national LHS.

## Acknowledgements

 The authors wish to thank their colleagues who provided input at different stages of developing the LCP framework including all co-authors of literature reviews.

## Ethical issues

 As a comprehensive, theory-informed approach, this methodological article did not require ethical approval.

## Conflicts of interest

 Authors declare that they have no conflicts of interest.

## Supplementary files


Supplementary file 1. Standards for Reporting Implementation Studies.
