# Supplementary material for: Learning Care Pathways Framework: A New Method to Implement, Learn, Replicate, and Scale up Care Pathways for and With the Patient
Source: Int J Health Policy Manag. 2025 Mar 11;14:8517. doi: 10.34172/ijhpm.8517 (PMC12032254; doi:10.34172/ijhpm.8517)
Supplement: Supplementary file 1 — Standards for Reporting Implementation Studies. [file ijhpm-14-8517-s001.pdf]

Article title: Learning Care Pathways Framework: A New Method to Implement, Learn, Replicate, and Scale up Care Pathways for and With the Patient

Journal name: International Journal of Health Policy and Management (IJHPM)

Authors' information: Jean-Baptiste Gartner[1,2,3,4,5,6,*], Célia Lemaire[7,8], André Côté[1,2,3,4,5,6]

[1]Département de management, Faculté des sciences de l'administration, Université Laval, Québec City, QC, Canada.

[2]Centre de recherche en gestion des services de santé, Université Laval, Québec City, QC, Canada.

[3]Centre de recherche de l'Institut Universitaire de Cardio-Pneumologie de Québec, Université Laval, Québec City, QC, Canada.

[4]Centre de recherche du CHU de Québec, Université Laval, Québec City, QC, Canada.

[5]VITAM, Centre de recherche en santé durable, Université Laval, Québec City, QC, Canada.

[6]Centre de recherche du CISSS de Chaudière-Appalaches, Université Laval, Québec City, QC, Canada.

[7]iaelyon School of Management, Université Lyon 3, Lyon, France.

[8]Institut Universitaire de France, Paris, France.

***Correspondence to:** Jean-Baptiste Gartner; Email: jean-baptiste.gartner@fsa.ulaval.ca

Citation: Gartner JB, Lemaire C, Côté A. Learning care pathways framework: a new method to implement, learn, replicate, and scale up care pathways for and with the patient. Int J Health Policy Manag. 2025;14:8517. doi:10.34172/ijhpm.8517

Supplementary file 1. Standards for Reporting Implementation Studies

This checklist has been adapted for use with this publication but follows in every respect the checklist from Table 1 Standards for Reporting Implementation Studies: the StaRI Checklist of items to be reported, in Pinnock H, Barwick M, Carpenter CR, Eldridge S, Grandes G, Griffiths CJ, et al. Standards for reporting implementation studies (StaRI) statement. *bmj*. 2017;356. https://doi.org/10.1136/bmj.i6795

**Checklist item**		**Implementation strategy**	**Intervention†**	**Information reported** Yes	No	**Line number(s)**
**Title**	1	Identification as an implementation study, and description of the methodology in the title and/or keywords		☒	☐	Title
**Abstract**	2	Identification as an implementation study, including a description of the implementation strategy to be tested, the evidence-based intervention being implemented, and defining the key implementation and health outcomes		☒	☐	Abstract
**Introduction**	3	Description of the problem, challenge, or deficiency in healthcare or public health that the intervention being implemented aims to address		☒	☐	1-15
	4	The scientific background and rationale for the implementation strategy (including any underpinning theory, framework, or model, how it is expected to achieve its effects, and any pilot work)	The scientific background and rationale for the intervention being implemented (including evidence about its effectiveness and how it is expected to achieve its effects)	☒	☐	40-53
**Aims and objectives**	5	The aims of the study, differentiating between implementation objectives and any intervention objectives		☒	☐	17-38
**Methods: description**	6	The design and key features of the evaluation (cross referencing to any appropriate methodology reporting standards) and any changes to study protocol, with reasons		☒	☐	325-341
	7	The context in which the intervention was implemented (consider social, economic, policy, healthcare, organisational barriers and facilitators that might influence implementation elsewhere)		☐	☒	
	8	The characteristics of the targeted "site(s)" (locations, personnel, resources, etc) for implementation and any eligibility criteria	The population targeted by the intervention and any eligibility criteria	☐	☒	
	9	A description of the implementation strategy	A description of the intervention	☒	☐	151-442
	10	Any subgroups recruited for additional research tasks, and/or nested studies are described		☐	☒	
**Methods: evaluation**	11	Defined pre-specified primary and other outcome(s) of the implementation strategy, and how they were assessed. Document any pre-determined targets	Defined pre-specified primary and other outcome(s) of the intervention (if assessed), and how they were assessed. Document any pre-determined targets	☒	☐	325-341
	12	Process evaluation objectives and outcomes related to the mechanism(s) through which the strategy is expected to work		☒	☐	325-341
	13	Methods for resource use, costs, economic outcomes, and analysis for the implementation strategy	Methods for resource use, costs, economic outcomes, and analysis for the intervention	☒	☐	325-341
	14	Rationale for sample sizes (including sample size calculations, budgetary constraints, practical considerations, data saturation, as appropriate)		☐	☒	

	15	Methods of analysis (with reasons for that choice)		☒	☐	325-341
	16	Any a priori subgroup analyses (such as between different sites in a multicentre study, different clinical or demographic populations) and subgroups recruited to specific nested research tasks		☐	☒	
**Results**	17	Proportion recruited and characteristics of the recipient population for the implementation strategy	Proportion recruited and characteristics (if appropriate) of the recipient population for the intervention	☐	☒	
	18	Primary and other outcome(s) of the implementation strategy	Primary and other outcome(s) of the intervention (if assessed)	☐	☒	
	19	Process data related to the implementation strategy mapped to the mechanism by which the strategy is expected to work		☒	☐	151-442
	20	Resource use, costs, economic outcomes, and analysis for the implementation strategy	Resource use, costs, economic outcomes, and analysis for the intervention	☐	☒	
	21	Representativeness and outcomes of subgroups including those recruited to specific research tasks		☐	☒	
	22	Fidelity to implementation strategy as planned and adaptation to suit context and preferences	Fidelity to delivering the core components of intervention (where measured)	☐	☒	
	23	Contextual changes (if any) which may have affected outcomes		☐	☒	
	24	All important harms or unintended effects in each group		☐	☒	
**Discussion**	25	Summary of findings, strengths and limitations, comparisons with other studies, conclusions and implications		☒	☐	443-485
	26	Discussion of policy, practice and/or research implications of the implementation strategy (specifically including scalability)	Discussion of policy, practice and/or research implications of the intervention (specifically including sustainability)	☒	☐	486-495
**General**	27	Include statement(s) on regulatory approvals (including, as appropriate, ethical approval, confidential use of routine data, governance approval), trial or study registration (availability of protocol), funding, and conflicts of interest		☐	☒	Title page

*Implementation strategy refers to how the intervention was implemented.
†Intervention refers to the healthcare or public health intervention that is being implemented.
